# Silent Myocardial Perfusion Abnormalities Detected by Stress Cardiovascular Magnetic Resonance in Antiphospholipid Syndrome: A Case-Control Study

**DOI:** 10.3390/jcm8071084

**Published:** 2019-07-23

**Authors:** Sophie I. Mavrogeni, George Markousis-Mavrogenis, Olga Karapanagiotou, Konstantinos Toutouzas, Panagiotis Argyriou, Stella Velitsista, George Kanoupakis, Dimitrios Apostolou, David Hautemann, Petros P. Sfikakis, Maria G. Tektonidou

**Affiliations:** 1 Onassis Cardiac Surgery Center, 17674 Athens, Greece; 2 First Cardiology Department, Medical School, National and Kapodistrian University of Athens, 11527 Athens, Greece; 3 Radiology Department, Mediterraneo Hospital, 16675 Athens, Greece; 4 Leiden University Medical Center, 2333 ZA Leiden, The Netherlands; 5 First Department of Propaedeutic Internal Medicine, Joint Rheumatology Program, Medical School, National and Kapodistrian University of Athens, 11527 Athens, Greece

**Keywords:** cardiovascular disease, autoimmune rheumatic diseases, antiphospholipid syndrome, cardiovascular magnetic resonance, ischemic cardiac disease, myocardial ischemia, myocardial fibrosis, late gadolinium enhancement

## Abstract

**Objective:** To examine the prevalence of silent myocardial ischemia and fibrosis in antiphospholipid syndrome (APS), using stress cardiovascular magnetic resonance (CMR). **Methods:** Forty-four consecutive APS patients without prior cardiac disease (22 primary APS, 22 systemic lupus erythematosus (SLE)/APS, mean age 44 (12.9) years, 64% women) and 44 age/gender-matched controls were evaluated using CMR at 1.5 T. Steady-state free precession imaging for function assessment and adenosine stress-CMR for perfusion-fibrosis evaluation were employed. The myocardial perfusion reserve index (MPRI), and myocardial fibrosis expressed as late gadolinium enhancement (LGE), were evaluated. Coronary angiography was indicated in patients with LGE. Associations with APS characteristics, classic cardiovascular disease (CVD) risk factors, high-sensitivity CRP (hs-CRP) and high-sensitivity Troponin (hs-TnT) levels were tested. All patients were followed up for 12 months. **Results:** Median MPRI was significantly lower in APS patients versus controls [1.5 (0.9–1.9) vs. 2.7 (2.2–3.2), *p* < 0.001], independently of any LGE presence. LGE was detected in 16 (36.3%) patients versus none of controls (*p* < 0.001); 12/16 were subsequently examined with coronary angiography and only two of them had coronary artery lesions. In multivariable analysis, none of the APS-related and classic CVD risk factors, or hs-CRP and hs-TnT covariates, were significant predictors of abnormal MPRI or LGE. At the twelve month follow-up, three (6.8%) patients experienced coronary artery disease, notably those with the lowest MPRI values. **Conclusions:** Abnormal MPRI and LGE are common in asymptomatic APS patients, independently so of any APS-related and classic CVD risk factors, or coronary angiography findings in cases with LGE. Stress-CMR is a valuable tool to detect silent myocardial ischemia and fibrosis in APS.

## 1. Introduction

Antiphospholipid syndrome (APS) is a rare systemic autoimmune disorder characterized by vascular thrombosis of large, medium or small vessels, pregnancy morbidity and persistently positive antiphospholipid antibodies (aPL), including lupus anticoagulant, anticardiolipin antibodies and/or anti–β2-glycoprotein I antibodies [1]. These antibodies may be detected individually or in combinations of two or three (double- or triple-positive aPL, respectively). APS may occur in its primary form (primary APS, PAPS) or in correlation with other autoimmune diseases, mainly systemic lupus erythematosus (SLE/APS) [2].

Heart involvement is one of the major complications in APS, including valve and ischemic heart disease [3,4,5]. In a multi-center European cohort of 1000 patients with APS, heart valve disease—mostly valve thickening and/or vegetation—was observed in 30% of patients, while ischemic heart disease manifesting mainly as myocardial infarction (MI), was demonstrated in 5.5% of patients [2]. MI was the most common cause of death after bacterial infections among patients with APS, referring to 19% of deaths in a 5-year follow-up period [2]. Other types of heart disease have also been reported in APS, including coronary vasospasm, known as variant (Prinzmetal’s) angina and syndrome X [6,7], myocardial ischemia associated with thrombotic cardiac microvasculopathy [8], and endomyocardial fibrosis due to coronary microcirculation defects [9].

Cardiovascular magnetic resonance imaging (CMR) is a non-invasive, non-ionizing radiation imaging modality that can assess cardiac geometry and function, myocardial perfusion and fibrosis [10,11]. Only one previous study used CMR in APS that showed a significantly higher prevalence of occult myocardial fibrosis associated with microvascular disease, expressed as late gadolinium enhancement (LGE), in 27 patients with APS compared to 81 healthy controls [12]. Using exercise or pharmacological stress with adenosine (stress CMR), a three- to four-fold increase in myocardial blood flow (MBF) can occur in healthy individuals. The ratio of maximum stress MBF after adenosine use to baseline rest is defined as a myocardial perfusion reserve index (MPRI) (10). The MPRI indicates the functional severity of a coronary lesion, and is of substantial additive value because an anatomic coronary stenosis does not necessarily correspond to a reduction of coronary blood flow. MPRI can distinguish between normal subjects and patients with coronary artery disease (CAD) of either macrovascular or microvascular etiology (lesions in epicardial coronary arteries or micro-circulation defects, respectively) [13,14,15].

Previous stress CMR studies by our group and others have demonstrated perfusion abnormalities by means of MPRI in patients with cardiac syndrome X [16], Raynaud’s phenomenon [17] and SLE [18], with a much higher sensitivity than conventional imaging modalities. The CE-MARC study showed also the superiority of CMR over single photon emission tomography (SPECT) for the detection of myocardial ischemia and fibrosis [19]. No comparison between APS patients and healthy controls has been carried out to date with regard to stress CMR findings [20]. 

Our aim was to examine myocardial perfusion defects using stress CMR in patients with PAPS and SLE/APS without known CAD, in comparison with age- and sex-matched healthy controls. We also evaluated potential associations between CMR findings and APS-related and classic CVD risk factors and coronary angiography findings.

## 2. Methods

### 2.1. Study Participants

Consecutive patients without any previous history of coronary artery disease (CAD) who met the updated Sapporo classification criteria for antiphospholipid syndrome (APS) [1] were recruited for participation in this study. Patients with SLE/APS fulfilled also the ACR classification criteria for systemic lupus erythematosus (SLE) [21]. Age- and sex-matched healthy controls were also recruited from a group of individuals participating in recreational sports. Exclusion criteria were prior atherosclerotic-origin cardiovascular disease (CVD), allergy to gadolinium, glomerular filtration rate (GFR) < 30 mL/min/1.75 m^2^, pregnancy or claustrophobia.

Demographic, clinical and laboratory characteristics of APS patients, and classic CVD risk factors, were recorded. Blood samples were collected at the time of examination and high-sensitivity CRP (hs-CRP) and high-sensitivity troponin T (hs-TnT) were measured by enzyme-linked immunosorbent assay (Elecsys 2010, Roche, Basel, Switzerland). All patients were followed up for 12 months, and clinically overt myocardial infarction (MI) or unstable angina was recorded based on physician adjudication. All participants provided informed consent and the protocol was approved by the Laikon Hospital Scientific Council.

### 2.2. Cardiovascular Magnetic Resonance Imaging Technique

All APS patients and matched healthy controls underwent a cardiovascular magnetic resonance (CMR) examination on a 1.5 Tesla scanner (Signa CV/i, GE Medical Systems) using ECG-triggered steady-state, free precession breath-hold cines (echo time (TE)/ repetition time (TR) 1.6/3.2 ms, flip angle 60) in long-axis planes and sequential 8 mm short-axis slices (3 mm gap) from the atrioventricular ring to the apex. Stress perfusion CMR was performed using a 140 mg/kg/min adenosine infusion for 4 min [10,22] and a 0.05 mmol/kg Gd-DTPA bolus (Schering) was administered during the first-pass perfusion study (IR balanced Turbo Field Echo, TR 2.8 ms, TE 1.38 ms, FA 45, slice thickness 8 mm, preparation pulse delay 200 ms). A rest perfusion study was performed using the same protocol. Late gadolinium enhancement (LGE) images were acquired 10 min after the intravenous administration of an additional 0.1 mmol/kg of Gd-DTPA. Images were acquired in short-axis planes using an inversion-recovery gradient echo sequence for fibrosis detection (3D-Turbo field echo sequence, TR 5.1 ms, TE 2.5 ms, FA 15, slice thickness 8 mm). Inversion times were adjusted to null normal myocardium (typically 320–440 ms; pixel size 1.7 × 1.4 mm). 

### 2.3. Cardiovascular Magnetic Resonance Analysis

CMR scans were independently analyzed by two experienced observers blinded to the clinical data (SM, DH), and a consensus was used for discordant findings. The intra- and inter-observer variability was 0.88 and 0.85, respectively. Ventricular volumes and function were measured for both ventricles using standard techniques and analyzed using specialized software [23,24]. Perfusion defects were assessed by both visual and parametric analysis. Quantification was performed using a delineation of endo and epicardial left ventricular (LV) borders throughout first-pass perfusion (MEDIS system, Leiden, The Netherlands). Stress and rest perfusion slopes were derived using Fermi-fitting of signal intensity vs time and normalized to the LV blood pool slope. Myocardial perfusion reserve index (MPRI) was calculated by dividing the median hyperemic MBF during stress by the median resting MBF [8]. Epicardial and endocardial contours of the LV myocardium for three short-axis slices (basal, mid and apical) were determined by the software, and manually corrected if needed to acquire intensity over time curves, which were used to measure the MPRI. The whole myocardial, subendocardial and subepicardial MPRI were calculated as the ratio of stress/rest relative perfusion up-slope, corrected for LV cavity up-slope. The subendocardial and subepicardial layers were automatically defined by the software as the inner and outer 50% of the wall thickness between the contoured myocardium. Finally, LGE images were assessed for midwall or subepicardial enhancement, compatible with microvascular disease [25,26] and for subendocardial or transmural enhancement in the distribution of a coronary artery, suggestive of MI [27]. In the latter case, diagnostic coronary angiography was offered to the corresponding patient, without taking other clinical findings into account (e.g., exercise testing).

### 2.4. Statistical Analysis

Statistical analyses were performed with Stata v.15 SE. The normality of continuous variables was visually determined using Q-Q plots and/or histograms. Normally distributed variables are presented as mean (standard deviation), not-normally distributed continuous variables are presented as median (interquartile range), and binary variables are presented as N (%).

Differences between APS patients and matched controls were investigated using paired-sample *t*-tests for continuous variables, sign tests for continuous not-normally distributed variables and McNemar’s test for binary variables. For intra-group (non-paired) comparisons, normally distributed variables were compared with independent-sample *t*-tests, not-normally distributed variables with Mann-Whitney U tests, and binary variables with chi-square tests. Linear regression analyses were used for multivariable corrections when investigating continuous outcomes. Statistical significance was considered for *p* ≤ 0.05. 

## 3. Results

Forty four patients with APS (22 with PAPS, 22 with SLE-APS) were included in the study (all Caucasian, 64% female, mean age: 44 ± 13 years, median disease duration: 12.0 (5.5, 21.0). One to one age- and sex-matching of 44 healthy controls without any history of cardiac disease was performed (mean age: 44 ± 11 years, 64% female). Baseline characteristics of all APS patients, and comparisons between those with PAPS and SLE/APS, are presented in Table 1. Patients with PAPS had a significantly higher prevalence of a previous stroke of non-atherosclerotic origin [5 (23%) vs. 0 (0%) *p* = 0.018] and recurrent thrombosis [12 (55%) vs. 5 (23%) *p* = 0.030], compared to SLE/APS patients. Additionally, PAPS patients with detectable TnT (above the 99th percentile upper reference limit of 13.9 ng/L) had significantly higher hs-TnT levels compared to those with SLE/APS and detectable TnT [19.5 (7.0–36.0) vs. 6.7 (4.3–9.0), *p* = 0.040], but the proportion of patients with hs-TnT below the detection limit did not differ significantly between the two groups [15 (68%) vs. 12 (55%), *p* = 0.35]. The median MPRI in the entire APS cohort was 1.48 (0.9, 1.9).

Descriptive statistics and comparisons between the CMR indices of APS patients and their matched controls are presented in Table 2. The LV volumes and ejection fractions did not differ significantly between the two groups. However, APS patients had a significantly lower median LV mass compared to matched controls [81.0 (65.5–98.5) vs. 121.5 (112.0–140.0), *p* < 0.001]. Median RVEDV and RVESV were also lower in APS patients compared to controls, but only RVEDV reached statistical significance [RVEDV: 109.0 (84.0–126.5) vs. 125.0 (120.0–150.0), *p* < 0.001; RVESV 38.5 (29.0–48.5) vs. 45.0 (40.0–48.0), *p* = 0.057]. There was a trend for higher median RVEF in APS patients compared to controls, but this also did not reach statistical significance (*p* = 0.079). 

**Figure 1:**
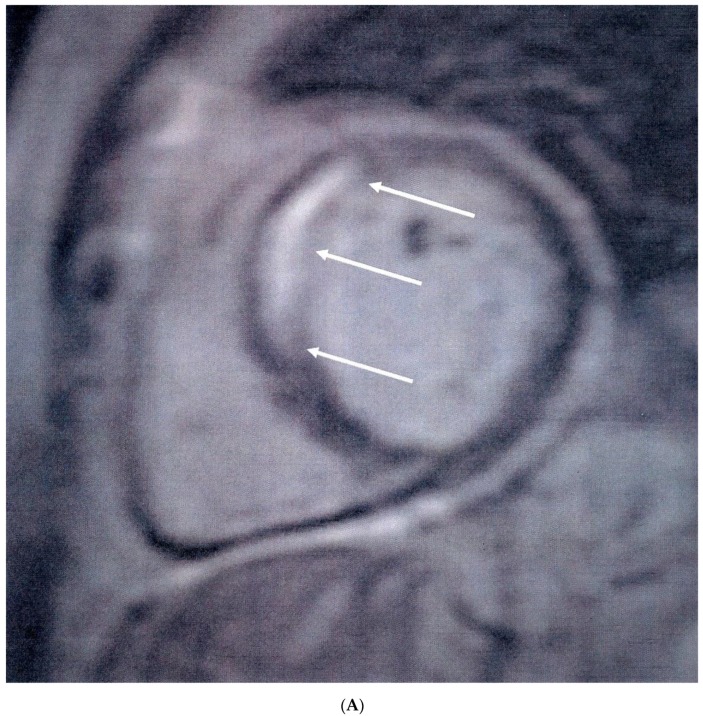

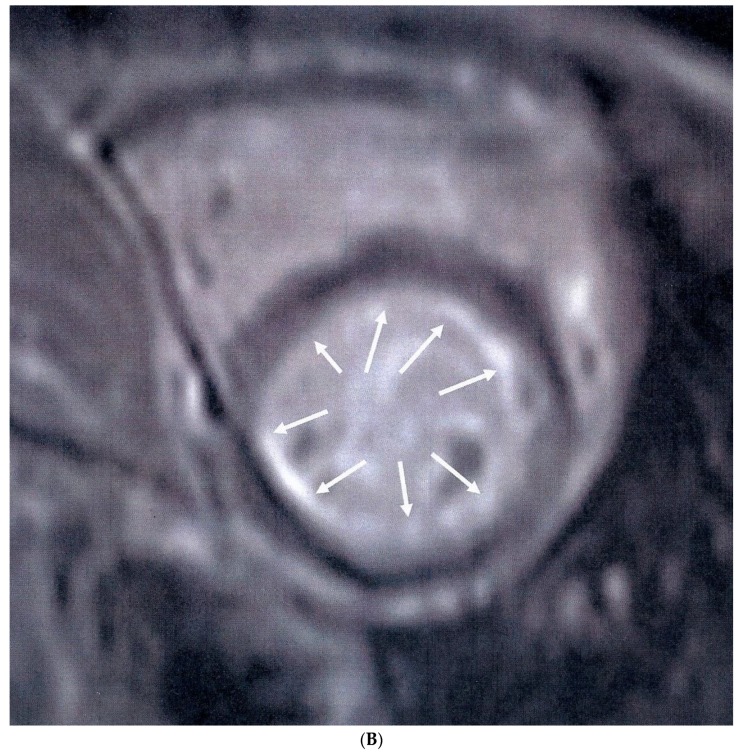
(**A**) Short-axis inversion recovery sequence showing septal myocardial scar (arrows), following the distribution of the left anterior descending artery, indicative of myocardial infarction. (**B**) Short-axis inversion recovery sequence showing diffuse subendocardial scar (arrows) due to microvascular disease.

Median MPRI was significantly lower in APS patients compared to healthy controls [1.48 (0.9–1.9) vs. 2.7 (2.2–3.2), *p* < 0.001]. Sixteen (36%) APS patients had visible areas of myocardial scar, expressed as LGE, compared to none of the healthy controls (*p* < 0.001). Myocardial scar following the distribution of coronary arteries was identified in nine (20%) patients (five in the anteroseptal and four in the inferolateral LV wall) (Figure 1A), while diffuse subendocardial fibrosis (DSF) (Figure 1B) was identified in seven (16%) patients. In patients with a positive LGE, the median LGE values expressed as percentage of LV mass were 4.5 (3.5–7.5). Coronary angiography was performed in 12 of the 16 APS patients with positive LGE, with macrovascular (obstructive) CAD identified only in two patients, one of which subsequently underwent angioplasty of the left anterior descending artery. Three of 10 patients with otherwise normal coronary angiography findings had abnormal LVEF values (<55%) and concomitant low MPRI values below the median of the APS group (<1.48).

When comparing patients with MPRI above and below the median value of the APS group (Table 3), no significant differences were identified in any demographic, clinical, laboratory and CMR characteristics, with the exception of obstetric APS and LVEF. The former was significantly less prevalent in patients with higher MPRI values [3 (15%) vs. 8 (33%), *p* = 0.029], and the latter was significantly higher in patients with higher MPRI values [61.5 (58.5, 65.0) vs. 64.5 (62.5, 68.0), *p* = 0.041], albeit still largely within normal limits (>55%). Additionally, more male patients had a lower MPRI, but this did not reach statistical significance [11 (46%) vs. 5 (75%), *p* = 0.153]. Finally, there was a trend for higher MPRI values in patients receiving treatment with corticosteroids compared to those not treated with corticosteroids, but this did not reach any statistical significance [9 (45%) vs. 5 (21%), *p* = 0.087]. A similar non-significant trend for higher MPRI was identified in patients treated with acetylsalicylic acid (ASA, Aspirin 100 mg daily) [12 (50%) vs. 5 (25%), *p* = 0.09]. No association with hydroxychloroquine use, or hsCRP and hsTnT levels was detected. Of the aforementioned variables, those with a *p*-value < 0.1 were included in a multivariable linear regression model for predicting MPRI values. Obstetric APS was replaced with female gender, as it would preclude the analysis of male patients. However, this did not lead to any statistically significant results (Table 4). No significant associations were also found between LGE and APS characteristics, classic CVD risk factors, or the hsCRP and hsTnT levels, in both descriptive (Table 5) and multivariate analysis.

At the twelve month follow-up, three patients (6.8%) experienced CAD complications (1 MI, and 2 unstable angina), importantly those with the lowest MPRI values (0.48, 0.56 and 0.58, respectively). However, meaningful statistical analyses could not be performed due to small numbers.

## 4. Discussion

This is the first study to our knowledge that evaluated the presence of silent myocardial ischemia and fibrosis by stress CMR in patients with APS. The most striking finding of our study is the detection of highly-reduced MPRI compared with age- and sex-matched healthy controls, irrespective of the presence of LGE. Myocardial fibrosis, expressed as LGE, was detected in one third of APS patients, of which only two patients had abnormal coronary angiography findings. These findings demonstrate that even in asymptomatic APS patients without evidence of fibrosis or macrovascular CAD detected by coronary angiography, silent myocardial ischemia may exist.

We also identified for the first time a significantly reduced LV mass in patients with APS compared to controls. Although it may be argued that the athletic activities followed by the healthy controls in our study might lead to LV mass increase, the actual difference between the groups is larger than what might reasonably be explained by exercise-induced LV hypertrophy alone [28]. On the other hand, little is known about myocardial mass changes preceding clinically overt cardiac involvement. According to currently published literature, reduced LV mass has been demonstrated in patients with rheumatoid arthritis, and was considered a predisposing factor for developing future heart failure [29]. Nevertheless, evidence is lacking about which factors can influence LV mass in APS apart from hemodynamics.

Myocardial perfusion defects in APS patients without prior CAD were previously examined by three studies using contrast echocardiography and scintigraphy with radionuclides [30], SPECT [31] and N-ammonia PET [32], respectively. Perfusion abnormalities were detected in 30%, 57.7% and 38.8% of 11, 26, and 18 patients tested, respectively. The limitations of these studies were the small number of included patients, the lack of comparison groups, and the use of imaging modalities of inferior diagnostic performance for myocardial perfusion assessment compared to CMR [14,22]. The only study that used CMR in APS showed a significantly higher prevalence of silent myocardial fibrosis compared to healthy controls [12]. No previous study examined silent myocardial ischemia using stress CMR imaging.

Our findings have important clinical implications for stratifying patients with APS at risk for heart disease. The early detection of reduced MPRI can be either due to macro- or micro-vascular CAD. If there is evidence of macro-vascular CAD, presenting as reduced MPRI and/or positive LGE following the distribution of coronary arteries, coronary angiography is indicated with subsequent standard interventional treatment, as needed. Microvascular disease on the other hand, presents as equally reduced MPRI in all myocardial territories with/or without DSF. In our study, we identified only two patients with macrovascular CAD, with the majority having microvascular CAD instead. Next to the aforementioned implications of low MPRI for microvascular CAD, the assessment of myocardial scar in asymptomatic APS patients has also important clinical implications for macrovascular CAD.

Firstly, it carries a prognostic value for the development of future arrhythmia [33] and/or heart failure [34] and secondly, it motivates and can justify further investigation with coronary angiography [34]. Finally, it may prompt initiation of specific medication use including ACE-inhibitors, β-adrenoreceptor blockers or aspirin [35].

These findings, in addition to the development of CAD events over a 12-month follow-up in three APS patients with the lowest MPRI at CMR, have also important therapeutic implications in APS. Given that almost all patients with APS were on adequate treatment with vitamin K antagonists, this finding raises questions about the presumed efficacy of currently employed anticoagulation strategies (as monotherapy) in criteria and non-criteria APS manifestations [36,37], and the potential need for additional cardioprotective treatment in patients with reduced MPRI [20]. In diabetic patients with abnormal MPRI without obstructive CAD, the use of antianginal agents was shown to improve microvascular ischemia [38], however, these effects in APS should be independently investigated in well-designed prospective studies. Lifestyle modification and aggressive monitoring and management of classic CVD risk factors are included in the general measures for the management of APS in adults [39,40].

Interestingly, no significant associations were detected in our study between abnormal MPRI or LGE and APS clinical and laboratory characteristics or traditional CVD risk factors. A previous study [12] showed a trend between myocardial scarring and APS features, such as disease duration and positivity for anti-β2-glycoprotein I antibodies, however, these associations did not reach statistical significance. In addition, we found no association between reduced MPRI and hs-TnT or hs-CRP levels. In a recent study of SLE patients [41], hs-TnT was significantly associated with myocardial edema detected by T2 mapping that was not evaluated by our stress CMR protocol. Patients receiving Aspirin treatment and those on corticosteroids tended to have higher MPRI values, but these trends did not reach statistical significance. However, inferences about these associations cannot be accurately drawn due to the relatively small number of our study population.

In addition to adenosine stress CMR use for the detection of silent perfusion abnormalities in APS, other strengths of the study are the comparison of CMR indices with coronary angiography findings in patients with abnormal LGE, and the 12-month follow-up of patients for CAD events. Our study has also some limitations. The relatively small number of patients might have precluded the identification of significant associations between MPRI and APS-related or classic CVD risk factors. Furthermore, novel CMR indices for the detection of diffuse myocardial edema and fibrosis (T1, T2 mapping and extracellular volume fraction quantification) [20] were not available in our department at the time of the CMR examination. In addition, coronary angiography was performed only in patients with an LGE presence. It could thus be argued that an unidentified macrovascular obstructive component in the coronary circulation might also be at work, given that microvascular disease is not the sole process leading to MPRI reductions without concomitant fibrosis.

In conclusion, silent myocardial ischemia and myocardial fibrosis are common in APS, independent of APS-related and classic CVD risk factors, hs-CRP and hs-TnT levels or coronary angiography findings. Our results support the diagnostic value of the CMR examination for the detection of silent micro- or macro-vascular CAD in APS. Early identification of ischemic heart lesions by CMR may motivate further cardiac investigation and an early initiation of cardioprotective treatment. In addition, our findings raise questions about the efficacy of currently-used therapeutic approaches for the prevention of CAD in APS, underlining the need for a re-evaluation of current practices by future studies.

## Figures and Tables

**Table 1:** Baseline demographic, clinical and cardiovascular magnetic resonance (CMR) characteristics of antiphospholipid syndrome (APS) patients.

Variable	All APS Patients	Primary APS	SLE/APS	*p*-Value
**Number of participants**	44.000	22.000	22.000	N/A
**Demographics**:				
Age (years)	44.0 (12.9)	45.9 (12.5)	42.0 (13.2)	0.320
Female gender	28 (64%)	12 (55%)	16 (73%)	0.210
BMI (kg/m^2^)	28.5 (5.2)	29.1 (4.3)	27.8 (6.1)	0.420
Disease Duration (Years)	12.0 (5.5, 21.0)	10.5 (6.0, 19.0)	14.5 (5.0, 21)	0.638
**APS Characteristics**:				
Obstetric APS (females only):				
Absent	17 (39%)	6 (27%)	11 (50%)	0.278
Present	11 (25%)	6 (27%)	5 (23%)	
Anticardiolipin antibodies	35 (80%)	17 (77%)	18 (82%)	0.710
Anti β2-glycoprotein I antibodies	29 (66%)	16 (73%)	13 (59%)	0.340
Lupus anticoagulant	35 (80%)	18 (82%)	17 (77%)	0.710
Double-positive aPL	15 (34%)	8 (36%)	7 (32%)	0.750
Triple-positive aPL	20 (45%)	11 (50%)	9 (41%)	0.540
**Cardiovascular risk factors**:				
Family History of CAD	4 (9%)	2 (9%)	2 (9%)	0.999
Smoking:				
Non-smoker	20 (45%)	11 (50%)	9 (41%)	
Smoker (past)	12 (27%)	5 (23%)	7 (32%)	0.770
Smoker (present)	12 (27%)	6 (27%)	6 (27%)	
Diabetes	2 (5%)	0 (0%)	2 (9%)	0.150
Hypertension	6 (14%)	4 (18%)	2 (9%)	0.380
Dyslipidaemia	6 (14%)	4 (18%)	2 (9%)	0.380
Number of CVD Risk Factors	2.0 (1.0, 2.0)	2.0 (1.0, 3.0)	1.5 (1.0, 2.0)	0.160
**Cardiovascular Medications**:				
ACE inhibitors	7 (16%)	3 (14%)	4 (18%)	0.680
Angiotensin receptor antagonists	3 (7%)	1 (5%)	2 (9%)	0.550
Calcium channel blockers	3 (7%)	2 (9%)	1 (5%)	0.550
Diuretics	2 (5%)	1 (5%)	1 (5%)	0.999
β-adrenoreceptor blockers	6 (14%)	4 (18%)	2 (9%)	0.380
Statins	5 (11%)	3 (14%)	2 (9%)	0.630
Anticoagulants	41 (93%)	22 (100%)	19 (86%)	0.073
Acetylsalicylic acid	17 (39%)	9 (41%)	8 (36%)	0.096
**Immunosupressive Medications**:				
Corticosteroids	14 (32%)	1 (5%)	13 (59%)	<0.001
Hydroxychloroquine	26 (59%)	8 (36%)	18 (82%)	0.002
Azathioprine	5 (11%)	4 (18%)	1 (5%)	0.154
Methotrexate	4 (9%)	1 (5%)	3 (14%)	0.290
Mycophenolate Mofetil	4 (9%)	0 (0%)	4 (18%)	0.036
Mycophenolic Acid	1 (2%)	0 (0%)	1 (5%)	0.310
**Previous Vascular Events**:				
Stroke	5 (11%)	5 (23%)	0 (0%)	0.018
Arterial thrombosis	19 (43%)	12 (55%)	7 (32%)	0.130
Venous thrombosis	30 (68%)	15 (68%)	15 (68%)	0.999
Recurrent thrombosis	17 (39%)	12 (55%)	5 (23%)	0.030
Recurrence on Anticoagulants	11 (26%)	7 (32%)	4 (19%)	0.340
**CMR Variables**:				
LV end diastolic volume (mL)	131.5 (110.0, 160.5)	131.5 (120.0, 172.0)	131.5 (104.0, 159.0)	0.310
LV end systolic volume (mL)	47.5 (38.5, 61.5)	49.5 (40.0, 69.0)	45.0 (33.0, 57.0)	0.250
LV ejection fraction (%)	63.5 (60.0, 67.0)	63.0 (60.0, 67.0)	64.0 (60.0, 67.0)	0.800
LV mass (g)	81.0 (65.5, 98.5)	86.0 (67.0, 106.0)	78.0 (60.0, 89.0)	0.150
RV end diastolic volume (mL)	109.0 (84.0, 126.5)	114.5 (89.0, 129.0)	103.5 (70.0, 116.0)	0.330
RV end systolic volume (mL)	38.5 (29.0, 48.5)	40.5 (32.0, 49.0)	36.0 (24.0, 46.0)	0.400
RV ejection fraction (%)	64.0 (59.5, 67.0)	63.0 (59.0, 66.0)	65.0 (60.0, 69.0)	0.450
LGE (present/absent)	16 (36%)	8 (36%)	8 (36%)	0.400
LGE as % LV mass (only if LGE is present)	4.5 (3.5–7.5)	6.5 (3.0, 15.0)	4.0 (3.5, 5.5)	0.290
Myocardial perfusion reserve index (MPRI)	1.5 (0.9, 1.9)	1.4 (0.9, 1.8)	1.5 (0.9, 2.1)	0.999
**Biomarkers**:				
High-sensitivity C-reactive protein (mg/L)	2.4 (1.2, 5.0)	1.7 (1.1, 5.0)	2.6 (1.2, 4.9)	0.760
High-sensitivity Troponin-T below lowest limit of detection	27 (61%)	15 (68%)	12 (55%)	0.350
High-sensitivity Troponin-T (pg/mL)–within detection range	7.8 (5.6, 19.6)	19.5 (7.0, 36.0)	6.7 (4.3, 9.0)	0.040

Values represent the median and interquartile range mean ± standard deviation (SD) for quantitative parameters and percent participants within each subgroup for qualitative characteristics. CMR = cardiovascular magnetic resonance; BMI = body mass index; APS = antiphospholipid syndrome; CAD = coronary artery disease; CVD = cardiovascular disease; LV = left ventricular; RV = right ventricular; LGE = late gadolinium enhancement, and here SLE = systemic lupus erythematosus.

**Table 2:** Comparison of CMR findings between APS patients and matched controls.

Parameters	Descriptive Statistics (APS Patients)	Descriptive Statistics (Matched Controls)	*p*-Value
Number of participants	44	44	N/A
LVEDV (mL)	131.5 (110.0, 160.5)	140.0 (123.0, 160.0)	0.628
LVESV (mL)	47.5 (38.5, 61.5)	51.0 (44.0, 63.5)	0.517
LVEF (%)	63.5 (60.0, 67.0)	63.0 (58.0, 66.0)	0.607
LV Mass (g)	81.0 (65.5, 98.5)	121.5 (112.0, 140.0)	<0.001
RVEDV (mL)	109.0 (84.0, 126.5)	125.0 (120.0, 150.0)	<0.001
RVESV (mL)	38.5 (29.0, 48.5)	45.0 (40.0, 48.0)	0.057
RVEF (%)	64.0 (59.5, 67.0)	60.0 (58.0, 64.0)	0.079
LGE	16 (36%)	0 (0%)	<0.001
MPRI	1.5 (0.9, 1.9)	2.7 (2.2, 3.2)	<0.001

These values represent the median and interquartile range. CMR = Cardiovascular Magnetic Resonance; APS = antiphospholipid syndrome; LVEDV = left ventricular end diastolic volume; LVESV = left ventricular end systolic volume; LVEF = left ventricular ejection fraction; LV = mass left ventricular mass; RVEDV = right ventricular end diastolic volume; RVESV = right ventricular end systolic volume; RVEF = right ventricular ejection fraction; LGE = late gadolinium enhancement; MPRI = myocardial perfusion reserve index.

**Table 3:** Comparison of patients with MPRI above and below the median of the group (=1.48).

Variable	MPRI below Median	MPRI above Median	*p*-Value
**Number of participants**	24	20	
**Demographics:**			
Age (years)	43.4 (13.3)	44.7 (12.6)	0.750
Female gender	13 (54%)	15 (75%)	0.153
BMI (kg/m^2^)	27.6 (5.9)	29.5 (4.2)	0.230
Disease Duration (Years)	14.0 (6.5, 21.0)	9.0 (4.0, 20.0)	0.395
**APS Characteristics:**			
Primary APS	13 (54%)	9 (45%)	0.540
Obstetric APS (females only):			
Absent	5 (21%)	12 (60%)	0.029
Present	8 (33%)	3 (15%)	
Anticardiolipin antibodies	18 (75%)	17 (85%)	0.410
anti β2-glycoprotein I antibodies	15 (63%)	14 (70%)	0.600
Lupus anticoagulant	20 (83%)	15 (75%)	0.500
Double-positive aPL	7 (29%)	8 (40%)	0.450
Triple-positive aPL	11 (46%)	9 (45%)	0.960
**Cardiovascular risk factors:**			
Family History of CAD	2 (8%)	2 (10%)	0.850
Smoking:			
Non-smoker	10 (42%)	10 (50%)	
Smoker (past)	6 (25%)	6 (30%)	0.610
Smoker (present)	8 (33%)	4 (20%)	
Diabetes	1 (4%)	1 (5%)	0.890
Hypertension	3 (13%)	3 (15%)	0.810
Dyslipidaemia	2 (8%)	4 (20%)	0.260
Number of CVD Risk Factors	2.0 (0.0, 2.0)	2.0 (1.0, 2.0)	0.300
**Cardiovascular Medications:**			
ACE inhibitors	4 (17%)	3 (15%)	0.880
Angiotensin receptor antagonists	1 (4%)	2 (10%)	0.440
Calcium channel blockers	2 (8%)	1 (5%)	0.660
Diuretics	2 (8%)	0 (0%)	0.190
β-Adrenoreceptor blockers	4 (17%)	2 (10%)	0.520
Statins	2 (8%)	3 (15%)	0.490
Anticoagulants	23 (96%)	18 (90%)	0.440
Acetylsalicylic acid	12 (50%)	5 (25%)	0.090
**Immunosupressive Medications:**			
Corticosteroids	5 (21%)	9 (45%)	0.087
Hydroxychloroquine	13 (54%)	13 (65%)	0.470
Azathioprine	2 (8%)	2 (8%)	0.488
Methotrexate	3 (13%)	1 (5%)	0.390
Mycophenolate Mofetil	2 (8%)	2 (10%)	0.850
Mycophenolic Acid	0 (0%)	1 (5%)	0.270
**Previous Vascular Events:**			
Stroke	2 (8%)	3 (14%)	0.490
Arterial thrombosis	10 (42%)	9 (45%)	0.820
Venous thrombosis	15 (63%)	15 (75%)	0.380
Recurrent thrombosis	9 (38%)	8 (40%)	0.870
Recurrence on Anticoagulants	5 (21%)	6 (32%)	0.420
**CMR Parameters:**			
Left ventricular end diastolic volume (mL)	129.0 (112.5, 158.5)	142.5 (110.0, 162.0)	0.800
Left ventricular end systolic volume (mL)	48.0 (39.5, 66.5)	47.0 (38.5, 60.5)	0.690
Left ventricular ejection fraction (%)	61.5 (58.5, 65.0)	64.5 (62.5, 68.0)	0.041
Left ventricular mass (g)	88.0 (66.5, 103.0)	76.0 (59.5, 84.0)	0.110
Right ventricular end diastolic volume (mL)	103.5 (79.0, 124.0)	115.5 (84.5, 129.5)	0.500
Right ventricular end systolic volume (mL)	38.5 (29.5, 48.5)	38.5 (28.5, 48.5)	0.970
Right ventricular ejection fraction (%)	62.5 (56.0, 66.0)	65.0 (62.0, 69.0)	0.073
LGE as % LV mass (only if LGE is present)	5.0 (3.0, 8.0)	4.0 (4.0, 7.0)	0.950
Late gadolinium enhancement (present/absent)	11 (46%)	5 (25%)	0.150
**Biomarkers:**			
High-sensitivity C-reactive protein (mg/L)	3.0 (0.8, 5.8)	2.2 (1.2, 2.7)	0.350
High-sensitivity Troponin-T below lowest limit of detection	14 (58%)	13 (65%)	0.650
High-sensitivity Troponin-T (pg/mL)–within detection range	7.3 (4.3, 21.3)	9.0 (5.8, 19.5)	0.490

Values represent the median and interquartile range mean ± standard deviation (SD) for quantitative parameters and percent participants within each subgroup for qualitative characteristics. MPRI = myocardial perfusion reserve index; BMI = body mass index; APS = antiphospholipid syndrome; CAD = coronary artery disease; CVD = cardiovascular disease.

**Table 4:** Multivariable linear regression analysis for predicting the MPRI values. Examined covariates were selecting on the basis of achieving a *p*-value < 0.1 between low and high MPRI.

Variable	Coefficient [95% Confidence Interval]	*p*-Value
Female gender	−0.0167 [−0.48, 0.45]	0.943
LVEF (%)	−0.0081 [−0.024, 0.041]	0.615
RVEF (%)	0.024 [−0.18, 0.067]	0.251
Corticosteroids	0.445 [−0.027, 0.918]	0.064
Acetylsalicylic acid	−2.651 [−0.67, 0.14]	0.196

MPRI = myocardial perfusion reserve index; LVEF = left ventricular ejection fraction; RVEF = right ventricular ejection fraction.

**Table 5:** Comparison of patients with and without late gadolinium enhancement (LGE).

Variable	LGE Absent	LGE Present	*p*-Value
**Number of participants**	28	16	
**Demographics:**			
Age (years)	44.4 (12.8)	43.1 (13.3)	0.750
Female gender	18 (64%)	10 (63%)	0.906
BMI (kg/m^2^)	29.3 (4.9)	27.0 (5.6)	0.170
Disease Duration (Years)	10.5 (6.5, 20.5)	14.0 (4.5, 22.0)	0.660
**APS Characteristics:**			
Primary APS	14 (50%)	8 (50%)	0.999
Obstetric APS (females only):			
Absent	9 (32%)	8 (50%)	0.298
Present	9 (32%)	2 (12.5%)	
Anticardiolipin antibodies	23 (82%)	12 (75%)	0.570
Anti β2-glycoprotein I antibodies	21 (75%)	8 (50%)	0.092
Lupus anticoagulant	21 (75%)	14 (88%)	0.320
Double-positive aPL	11 (39%)	4 (25%)	0.336
Triple-positive aPL	13 (46%)	7 (44%)	0.860
**Cardiovascular risk factors:**			
Family History of CAD	2 (7%)	2 (13%)	0.550
Smoking:			
Non-smoker	10 (36%)	10 (63%)	
Smoker (past)	11 (39%)	1 (6%)	0.054
Smoker (present)	7 (25%)	5 (31%)	
Diabetes	1 (4%)	1 (6%)	0.680
Hypertension	5 (18%)	1 (6%)	0.280
Dyslipidaemia	4 (14%)	2 (13%)	0.870
Number of CVD Risk Factors	2.0 (1.0, 2.0)	1.0 (0.0, 2.0)	0.029
**Cardiovascular Medications:**			
ACE inhibitors	3 (11%)	4 (25%)	0.210
Angiotensin receptor antagonists	3 (11%)	0 (0%)	0.170
Calcium channel blockers	2 (7%)	1 (6%)	0.910
Diuretics	0 (0%)	2 (13%)	0.056
β-Adrenoreceptor blockers	2 (7%)	4 (25%)	0.097
Statins	4 (14%)	1 (6%)	0.420
Anticoagulants	26 (93%)	15 (94%)	0.910
Acetylsalicylic acid	12 (43%)	5 (31%)	0.450
**Immunosupressive Medications:**			
Corticosteroids	9 (32%)	5 (31%)	0.950
Hydroxychloroquine	16 (57%)	10 (63%)	0.730
Azathioprine	2 (%)	1 (%)	0.910
Methotrexate	3 (11%)	1 (6%)	0.620
Mycophenolate Mofetil	0 (0%)	1 (6%)	0.180
Mycophenolic Acid	2 (7%)	2 (13%)	0.550
**Previous Vascular Events:**			
Stroke	4 (%)	1 (%)	0.420
Arterial thrombosis	14 (50%)	5 (31%)	0.230
Venous thrombosis	18 (64%)	12 (75%)	0.460
Recurrent thrombosis	12 (43%)	5 (31%)	0.450
Recurrence on Anticoagulants	7 (26%)	4 (25%)	0.950
**CMR Parameters:**			
Left ventricular end diastolic volume (mL)	126.5 (104.5, 147.5)	158.5 (123.5, 185.5)	0.071
Left ventricular end systolic volume (mL)	47.5 (36.5, 55.0)	52.0 (41.5, 80.5)	0.120
Left ventricular ejection fraction (%)	64.0 (61.0, 66.5)	61.0 (53.0, 67.5)	0.240
Left ventricular mass (g)	77.0 (61.5, 95.0)	85.5 (67.0, 107.0)	0.280
Right ventricular end diastolic volume (mL)	110.0 (84.0, 126.5)	104.5 (80.0, 127.0)	0.970
Right ventricular end systolic volume (mL)	38.5 (28.5, 49.5)	38.5 (30.0, 46.5)	0.760
Right ventricular ejection fraction (%)	65.0 (60.5, 68.5)	62.5 (55.0, 65.5)	0.092
Biomarkers:			
High-sensitivity C-reactive protein (mg/L)	2.2 (1.1, 3.3)	3.5 (1.2, 5.6)	0.407
High-sensitivity Troponin-T below lowest limit of detection	18 (64%)	9 (56%)	0.600
High-sensitivity Troponin-T (pg/mL)–within detection range	7.3 (5.6, 18.4)	9.0 (4.6, 36.0)	0.380

These values represent median and interquartile ranges mean ± standard deviation (SD) for the quantitative parameters and percent participants within each subgroup for qualitative characteristics. CMR = cardiovascular magnetic resonance; BMI = body mass index; APS = antiphospholipid syndrome; CAD = coronary artery disease; CVD = cardiovascular disease.

## Abbreviations

APS	antiphospholipid syndrome
aPL	antiphospholipid antibodies
CMR	cardiovascular magnetic resonance
CAD	coronary artery disease
CVD	cardiovascular disease
MPRI	Myocardial perfusion reserve index
LGE	late gadolinium enhancement
MBF	myocardial blood flow
LV	left ventricular
LVEDV	left ventricular end diastolic volume
LVESV	left ventricular end systolic volume
LVEF	left ventricular ejection fraction
RVEDV	right ventricular end diastolic volume
RVESV	right ventricular end systolic volume;
RVEF	right ventricular ejection fraction
hs-CRP	high-sensitivity CRP
hs-TnT	high-sensitivity troponin T
